# Effectiveness of grey and green engineered solutions for protecting the low-lying muddy coast of the Chao Phraya Delta, Thailand

**DOI:** 10.1038/s41598-022-24842-x

**Published:** 2022-11-28

**Authors:** Warit Charoenlerkthawin, Komkrit Bidorn, William C. Burnett, Jun Sasaki, Balamurugan Panneerselvam, Butsawan Bidorn

**Affiliations:** 1 Department of Water Resources Engineering, Chulalongkorn University, Bangkok, 10330 Thailand; 2 WISE Research Unit, Chulalongkorn University, Bangkok, 10330 Thailand; 3 Department of Earth, Ocean and Atmospheric Science, Florida State University, Tallahassee, FL 32306 USA; 4 Department of Socio-Cultural Environmental Studies, The University of Tokyo, Kashiwa, 277-8563 Japan; 5 Department of Community Medicine, Saveetha Medical College, SIMATS, Chennai, 602-105 India

**Keywords:** Civil engineering, Environmental impact

## Abstract

Coastal protection measures can be categorized into grey and green solutions in terms of their ecosystem impacts. As the use of grey solutions has become a serious issue due to environmental consequences during the last few decades, green/nature-based solutions have become prioritized. This study evaluates the effectiveness of grey and green solutions applied along the eastern Chao Phraya Delta (ECPD) based on historical shoreline change analysis and coastal observations using Light Detection and Ranging technology. The results from shoreline analysis indicate that nearshore breakwaters installed 100–250 m from the shoreline have successfully reclaimed the coastline with a sedimentation rate of 17–23 cm/y. Meanwhile, sand-sausage-submerged breakwaters were ineffective at stabilizing the coastline during 2002–2010 due to land subsidence. With a low subsidence rate, the rubble-mound-submerged breakwaters can reduce the shoreline retreat rate with a vertical deposition rate of about 5 cm/y. In contrast, use of a bamboo fence, a green solution widely used along muddy coasts, traps sediment at a rate of less than 1.3 cm/y and typically lasts only for 2–3 years after installation. Decomposed bamboo causes environmental degradation so local communities disapprove of the approach. Results reveal that grey solutions are more effective for stabilizing the ECPD coastline and result in less coastal environmental impact than the nature-based solution using a bamboo fence.

## Introduction

During the past century, significant coastal degradation due to shoreline retreat has been reported worldwide^1–11^. Shoreline recession found around the world is dominated by several factors such as sea-level change^6–8^, storm surge^12^, land subsidence^13,14^, and coastal development^15^. Since shoreline change can cause negative impacts not only on human lives and societies but also on coastal environments, many coastal protection measures, either grey/hard or green/nature-based solutions, have been applied to mitigate the adverse effects^16–18^. However, unanticipated impacts due to coastal protection measures have been found in many regions during the last decades leading to unsustainable coastal protection and development. Up to present, the appropriateness of using grey or green measures to provide sustainable coastal development, which is a part of the United Nations Sustainable Development Goals^14^, is still debated.

Grey/hard solutions such as groins, breakwaters, and seawalls have a long history of stabilizing the shoreline for more than half a century^17,19^. Even though these conventional structures were designed based on scientific theories, they are now considered a hard solution or “grey” infrastructure in terms of environmental consequences^16,17,20–22^. It was reported that hard solutions altered coastal morphology, resulting in changes in ecological systems^20^. For example, foreshore structures (seawalls and sea dikes) can result in adverse long-term erosion on the adjacent coastline ^16,17,20^. They can also obstruct tides causing intertidal area loss. “Green”/nature-based solutions have recently been promoted to mitigate the detrimental consequences of hard solutions^16,17,23^. These green solutions are divided into three categories: (1) hybrid solutions, a combination of coastal habitat with hard solutions; (2) environmental-friendly grey solutions, the hard solutions designed to reduce ecosystem service losses; and (3) soft solutions, typically with no structure used^16,23,24^. Recently, using green/nature-based solutions has been encouraged as a priority over hard solutions to achieve sustainable coastal development worldwide. However, the effectiveness of grey and green solutions on coastal preservation and ecological impacts on different coastal environments has rarely been documented.

The Chao Phraya Delta (CPD), the largest delta and low-lying muddy coast in Thailand, has experienced shoreline recession for over six decades due to relative sea-level rise^25–30^. With an average shoreline retreat rate during 1996–2002 of more than − 16 m/y, the CPD has become one of the world’s shoreline retreat hot spots^31^. As the CPD hosts the capital city (Bangkok), including the major industrial area (Samut Prakan province) of Thailand, many coastal protection measures have been applied along the CPD coastline for almost three decades to stabilize this major socioeconomic area^32,33^. Hard solutions (seawalls, revetments, nearshore breakwaters, and geotextile sandbags) were used in the early stages of coastal protection. Meanwhile, green/nature-based solutions such as mangrove reforestation and bamboo fences have been applied over the last two decades. However, the effectiveness of each solution has not yet been systematically monitored. Up to the present, both grey and green solutions have been randomly applied along this muddy coastline. Therefore, the sustainability of the coastal development plan in this area is still in question.

**Figure 1:**
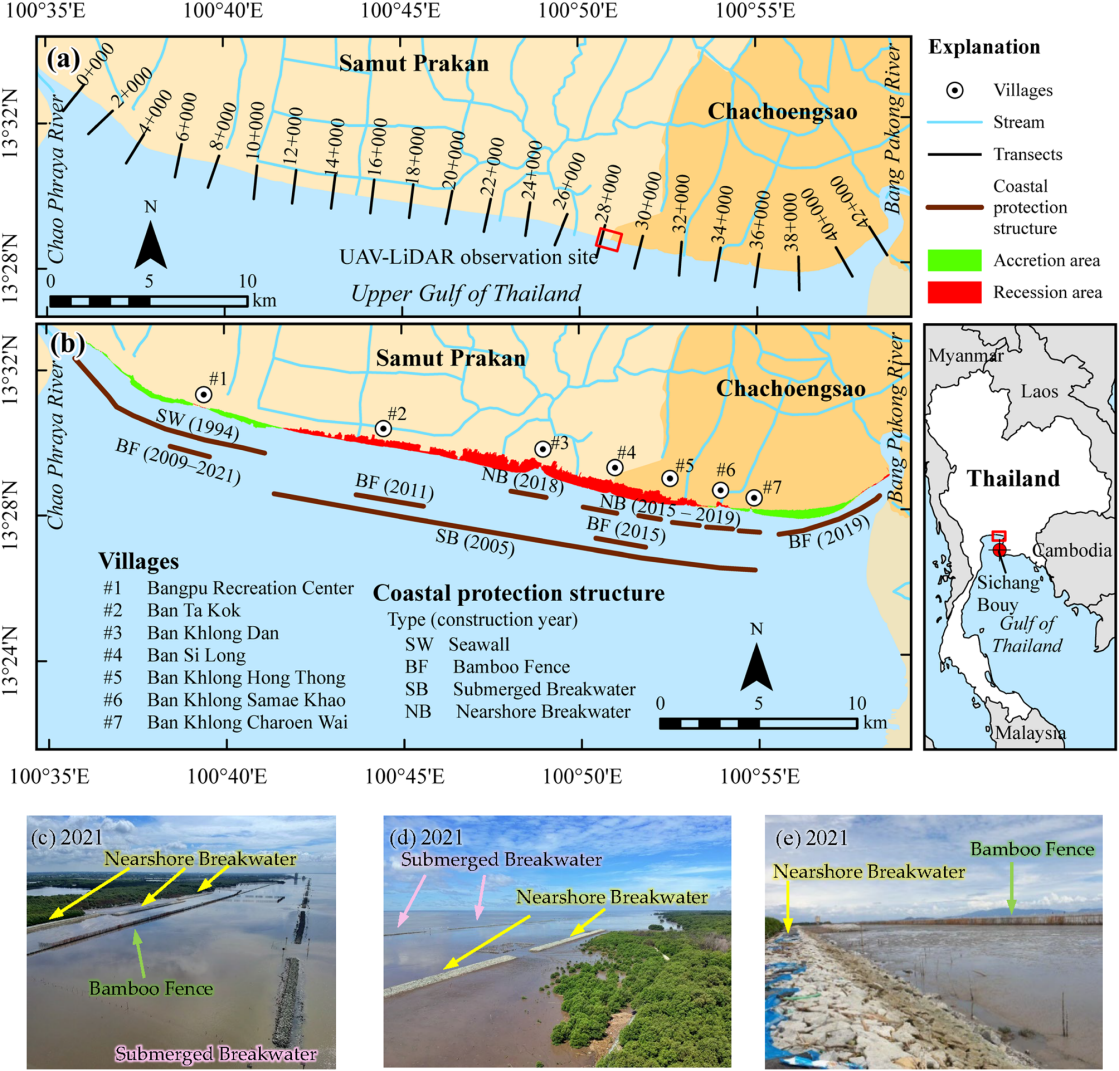
(**a**) Shoreline change measurement stations used in this study. The red box shows the LiDAR survey area. (**b**) Types of coastal structures and their alignment along the ECPD coastline. Red and green patches along the shore represent land loss and land accretion during 1954–2002. The maps were generated using ArcMap software version 10.6 ( © ESRI, https://desktop.arcgis.com). (**c**) The location of nearshore breakwaters, bamboo fences, and submerged breakwaters placed east of Ban Si Long. (**d**) The location of nearshore and submerged breakwaters placed west of Ban Si Long. (**e**) The crest of nearshore breakwaters above the seabed is typically lower than that of bamboo fences (The images **c**–**e** were taken by author).

We summarize here the historical rate of shoreline changes before and after coastal protection measures (submerged and nearshore breakwaters, bamboo fences) that were employed along the muddy coast of the eastern Chao Phraya Delta (ECPD; Fig. 1). In addition, we assessed the short-term effectiveness of grey and green solutions on shoreline stabilization using LiDAR technology. The results of this study should facilitate sustainable coastal development and planning, not only along the CPD muddy coast, but also for other low-lying deltas experiencing high rates of shoreline recession.

## Results

**Figure 2:**
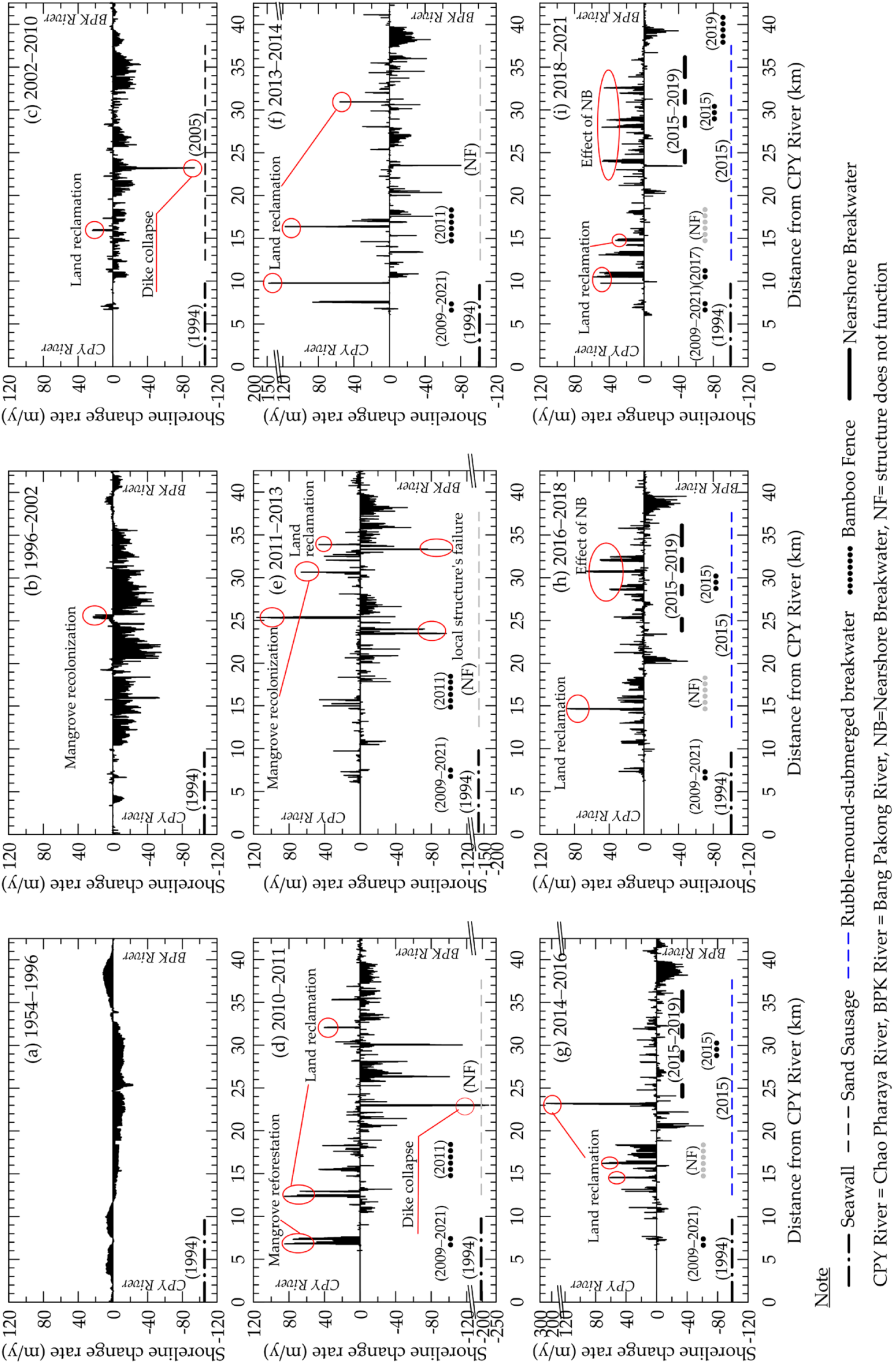
Long-term and short-term (interannual) shoreline change rates on the eastern Chao Phraya Delta (ECPD) coastline during 1954–2021.

The shoreline change analysis along the coast between the Chao Phraya (CPY) and Bang Pakong (BPK) Rivers between 1954 and 2021 is shown in Fig. 2, and the statistical results are summarized in Table 1. Details concerning the study area and methodologies are provided in a later section (“Methods and Study Area”). The shoreline analysis has been divided into six periods to study the effectiveness of these coastal protection measures. Table 2 provides information on shoreline change rates along the protected coastlines for each coastal structure. The pattern of shoreline movement for each period is described below.

**Table 1:** Results of both long-term and short-term (interannual) shoreline change on the eastern Chao Phraya coast.

Parameters	1954–1996	1996–2002	2002–2010	2010–2011	2011–2013	2013–2014	2014–2016	2016–2018	2018–2021
Mean NSM (m)	− 77 ± 5	− 65 ± 5	− 49 ± 1.2	− 5 ± 1.2	− 4 ± 1.2	− 3 ± 1.2	3 ± 1.1	8 ± 1.1	12 ± 1.2
Min NSM (m)	− 953 ± 5	− 340 ± 5	− 747 ± 1.2	− 331 ± 1.2	− 124 ± 1.2	− 77 ± 1.2	− 124 ± 1.1	− 141 ± 1.1	− 105 ± 1.2
Max NSM (m)	482 ± 5	138 ± 5	186 ± 1.2	126 ± 1.2	142 ± 1.2	139 ± 1.2	496 ± 1.1	250 ± 1.1	139 ± 1.2
Erosion area (ha)	694 ± 0.1	261 ± 0.1	186 ± 0.1	22 ± 0.1	15 ± 0.1	10 ± 0.1	18 ± 0.1	20 ± 0.1	8 ± 0.1
Accretion area (ha)	388 ± 0.1	12 ± 0.1	13 ± 0.1	13 ± 0.1	8 ± 0.1	5 ± 0.1	23 ± 0.1	34 ± 0.1	30 ± 0.1
Erosion area rate (ha/y)	17 ± 0.1	43 ± 0.1	23 ± 0.1	22 ± 0.1	8 ± 0.1	10 ± 0.1	9 ± 0.1	10 ± 0.1	3 ± 0.1
Accretion area rate (ha/y)	9 ± 0.1	2 ± 0.1	2 ± 0.1	13 ± 0.1	4 ± 0.1	5 ± 0.1	12 ± 0.1	17 ± 0.1	10 ± 0.1
Accretion (%)	46	12	9	13	14	5	18	27	23
Retreat (%)	53	69	56	25	22	17	18	17	10
Stable (%)	1	19	35	62	64	78	64	56	68
*Shoreline change rate using EPR (m/y)*									
Mean shoreline change rate	− 2 ± 0.1	− 11 ± 0.8	− 6 ± 0.1	− 3 ± 1.2	− 4 ± 0.6	− 3 ± 1.2	1 ± 0.6	3 ± 0.6	5 ± 0.4
Maximum accretion rate	12 ± 0.1	23 ± 0.8	23 ± 0.1	85 ± 1.2	117 ± 0.6	145 ± 1.2	245 ± 0.6	89 ± 0.6	59 ± 0.4
Mean accretion rate	5 ± 0.1	5 ± 0.8	5 ± 0.1	15 ± 1.2	10 ± 0.6	21 ± 1.2	14 ± 0.6	12 ± 0.6	14 ± 0.4
Maximum retreat rate	− 23 ± 0.1	− 56 ± 0.8	− 94 ± 0.1	− 224 ± 1.2	− 102 ± 0.6	− 80 ± 1.2	− 61 ± 0.6	− 50 ± 0.6	− 44 ± 0.4
Mean retreat rate	− 8 ± 0.1	− 17 ± 0.8	− 9 ± 0.1	− 15 ± 1.2	− 13 ± 0.6	− 12 ± 1.2	− 11 ± 0.6	− 9 ± 0.6	− 7 ± 0.4

NSM = net shoreline movement, EPR = end point rate.

**Table 2:** Summary of coastal structure details and the average shoreline change rate pre-and post-construction.

Types of coastal protection structures								
Parameters	Submerged breakwater (Sand sausage)	Submerged breakwater (Rubble mound)	Nearshore breakwater	Bamboo Fence				
Number of structures	112	112	28	–				
Distance from the initial shoreline (m)	6–558	100–800	40–280	50–200				
Average distance (m)	233	304	132	–				
Length of structure (m)	87–230	87–230	45–245	100–4140				
Average length (m)	166	166	191	–				
Height of structure (m)	1	1	2–3	–				
Construction year	2004–2005	2014–2015	2015–2019	2009–2021				
*Percentages of breakwater respond to the shoreline after construction (%)*								
Accretion	6%	57%	82%	31%				
Erosion	88%	28%	0%	26%				
Stable	6%	15%	18%	43%				

Average shorelines change rate pre-and post-construction								
	Submerged breakwater (Sand sausage)		Submerged breakwater (Rubble mound)		Nearshore breakwater		Bamboo Fence	
Parameters	Pre- (1996–2002)	Post- (2002–2010)	Pre- (2010–2014)	Post- (2016–2021)	Pre- (2010–2014)	Post- (2016–2021)	Pre- (2010–2014)	Post- (2014–2021)
Avg. shorelines change rate (m/y)	−14 ± 0.78	−7 ± 0.15	−2 ± 0.23	2 ± 0.23	−1 ± 0.23	10 ± 0.23	−2 ± 0.23	4 ± 0.23
Max. shoreline accretion rate (m/y)	20 ± 0.78	9 ± 0.15	19 ± 0.23	19 ± 0.23	10 ± 0.23	31 ± 0.23	2 ± 0.23	12 ± 0.23
Avg. shoreline accretion rate (m/y)	3 ± 0.78	2 ± 0.15	3 ± 0.23	4 ± 0.23	4 ± 0.23	11 ± 0.23	1 ± 0.23	6 ± 0.23
Max. shoreline retreat rate (m/y)	−43 ± 0.78	−60 ± 0.15	−23 ± 0.23	-13 ± 0.23	−16 ± 0.23	0 ± 0.23	−10 ± 0.23	−3 ± 0.23
Avg. shoreline retreat rate (m/y)	−16 ± 0.78	−8 ± 0.15	−5 ± 0.23	-2 ± 0.23	−4 ± 0.23	0 ± 0.23	−4 ± 0.23	−2 ± 0.23

Avg. = average; Max. = Maximum.

### Shoreline change rate during 1954–1996 (prior to coastal protection projects)

Before the series of coastal protection structures were constructed along the ECPD coastline, severe erosion was reported in several locations, especially along the coastlines of Bangkok and Samut Prakan provinces^34^. Based on the analysis of ECPD shoreline changes between 1954 and 1996 (Table 1), about 46% of the total coastline advanced seaward with an average rate of 5 m/y. The accretion along the shoreline mainly occurred near the CPY and BPK Rivers (Fig. 2a). The shoreline near the CPY River mouth advanced by an average of 4 m/y, while the coastline near the BPK River mouth migrated seaward at an average rate of 5 m/y. The net land accretion area was approximately 388 ha (9 ha/y). Over this same period, almost 53% of the total shoreline, mainly located along the mid-portion of the coastline, underwent significant shoreline recession. The shoreline retreat rate averaged − 8 m/y resulting in 695 ha of land loss over four decades (16.5 ha/y). The maximum setback of the shoreline took place at the mid-point of the coastline, at Ban Khlong Dan (#3 in Fig. 1b). The remaining 1% of the coastline was considered stable as the shoreline change rate was less than the measurement uncertainty (± 1 m/y).

### Shoreline change due to seawall

In 1994, an approximately 10-km long seawall was constructed along the shore near the CPY River mouth, covering 23% of the ECPD coastline. However, the data of Fig. 2b and Table 1 indicate that the recession along the ECPD coastline during 1996–2002 was worse than during the earlier period, 1954–1996. The seawall stabilized about 19% of the shoreline. Even though the average rate of shoreline advance was similar to that during 1954–1996, the accretion shoreline plummeted to only 12%, resulting in a land accretion rate of 2 ha/y. The maximum rate of shoreline advance occurred near the middle of the ECPD coastline due to mangrove recolonization in some abandoned shrimp ponds. However, the average shoreline recession increased to about − 17 m/y resulting in a land loss of approximately 43 ha/y. The maximum shoreline retreat increased dramatically to − 56 m/y in the mid-segment of the ECPD coastline. In addition, the shorelines near both river mouths shifted landward.

### Shoreline change due to geocontainer-submerged breakwater (“sand sausage”)

Responding to the severe shoreline retreat between the CPY and BPK River mouths, the Marine Department placed geo-sand containers or sand sausages, which have successfully protected sandy beaches in many countries^10,35–37^. This approach formed a submerged breakwater along 25 km of eroded coastline (Figs. 1a, b) during 2004–2005. Figure 2c shows that shoreline recession persisted along the mid-portion of the coast after the placement of these sand sausages for five years and accounted for 56% of the total coastline. However, the average shoreline recession was reduced from − 17to − 9 m/y. The very high shoreline retreat found at the middle of the coastline was caused by a shrimp pond’s dike collapse. Results from short-term (interannual) shoreline change analysis (1–3 year; Figs. 2d–f) revealed that the stable shoreline apparently increased to 62–78% of the ECPD shoreline during 2010–2014. But a remarkable rate of shoreline retreat occurred along the protected area due to the collapse of private coastal protection structures as well as shrimp pond dikes. Meanwhile, a rapid shoreline advance was found between 2010 and 2014 due to land reclamation using revetments.

Comparison of shoreline change rates between pre-and post-construction of sand sausages along the 25-km protected area (Table 2) shows that about 6% of the protected coastline was stable after the sand sausages were placed in 2005. Only 6% of the coastline moved seaward, with an average shoreline advance rate reduced from 3 to 2 m/y. About 88% of the ECPD coastline still underwent shoreline recession, but the average rate of shoreline retreat was only half of that before construction. The maximum shoreline retreat rate, which increased from − 43 to − 60 m/y, occurred near the mid-point of the ECPD coastline due to collapse of shrimp pond’s dikes.

### Shoreline change due to rubble-mound-submerged breakwater

Since almost 90% of the protected coastline still retreated after installing the sand sausages, the Marine Department placed rubble-mound-submerged breakwaters on top of the existing sand sausages during 2014–2015 because the sand sausages were damaged after 2–5 years of their installation. Moreover, the depth of the sand sausage crest was generally lower than the initial condition due to the high land subsidence rate along this muddy coast. After the construction of 1-m high rubble mound breakwaters, the percentage of accretion coastline grew from 5 to 27% during 2016–2021 (Figs. 2g–i).

Table 2 indicates that the accretion shoreline increased from 6 to 57% after the maintenance of submerged breakwater using rubble mound, and the eroded shoreline decreased to 28%. Even though shoreline accretion was slightly different between pre-and post-construction, shoreline retreat abated with average and maximum rates after construction reducing to − 2 and − 13 m/y, respectively.

### Shoreline change due to nearshore breakwater

Even though the percentage of shoreline recession along the ECPD coastline between 2014 and 2018 decreased to 17–18% of the total coastline, several coastal communities were still affected by shoreline retreat. During 2015–2019, the local government constructed 28 nearshore breakwaters to protect major communities located near the entrance of drainage canals (villages #3–#7 in Figs. 1b-e). With a height of 2–3 m (Table 2), the nearshore breakwaters’ crest is generally higher than average high tidal level, and the length of the structure was longer than submerged breakwaters on average. During 2018–2021 (after the construction of nearshore breakwaters; Fig. 2i), the percentage of stable shorelines noticeably increased to 68%. Although the percentage of shorelines accreting slightly reduced to 23%, high accretion rates of 41–48 m/y were observed along the coastline where the nearshore breakwaters were constructed. The shoreline retreat accounted for only 10% of the total coastline, and the average shoreline retreat rate was slightly decreased to − 7 m/y. Table 2 shows that 82% of the protected coastline moved seaward with an average accretion rate increased from about 4 m/y to 11 m/y, higher than after the construction of submerged breakwaters. The remaining 18% of the protected area was stabilized by nearshore breakwaters, and no significant erosion behind the nearshore breakwaters was observed.

### Shoreline change due to bamboo fence

Bamboo fences, considered green structures, were installed along the ECPD coastline during 2009–2021 by the Department of Marine and Coastal Resources (DMCR). Typically, the construction of bamboo fences has no specific technical design. The bamboo fences were constructed at 50–200 m from an initial shoreline in front of the eroded coastline. The bamboo fences are generally at least 3 m high above the seabed, and the length of the fences depends on the length of the protected coastline in each area (Table 2). After the bamboo fence was installed, about 43% of the protected area was stable, and the shoreline accretion accounted for 31%. An average accretion rate of 5.5 m/y was higher than that before the bamboo fences installation, as shown in Table 2. The maximum accretion rate was observed near the CPY River mouth (#1 in Fig. 1b; Figs. 2d, f), where mangrove reforestation has been promoted since 2009. However, about 26% of the protected coastline still underwent shoreline retreat, but the average shoreline retreat rate was reduced to − 1.6 m/y. A high rate of shoreline retreat behind the bamboo fence was observed near the BPK River mouth (Fig. 2i), and the shoreline has continued to shift landward up to present.

### Effectiveness of coastal protection structures using remote sensing technique

**Figure 3:**
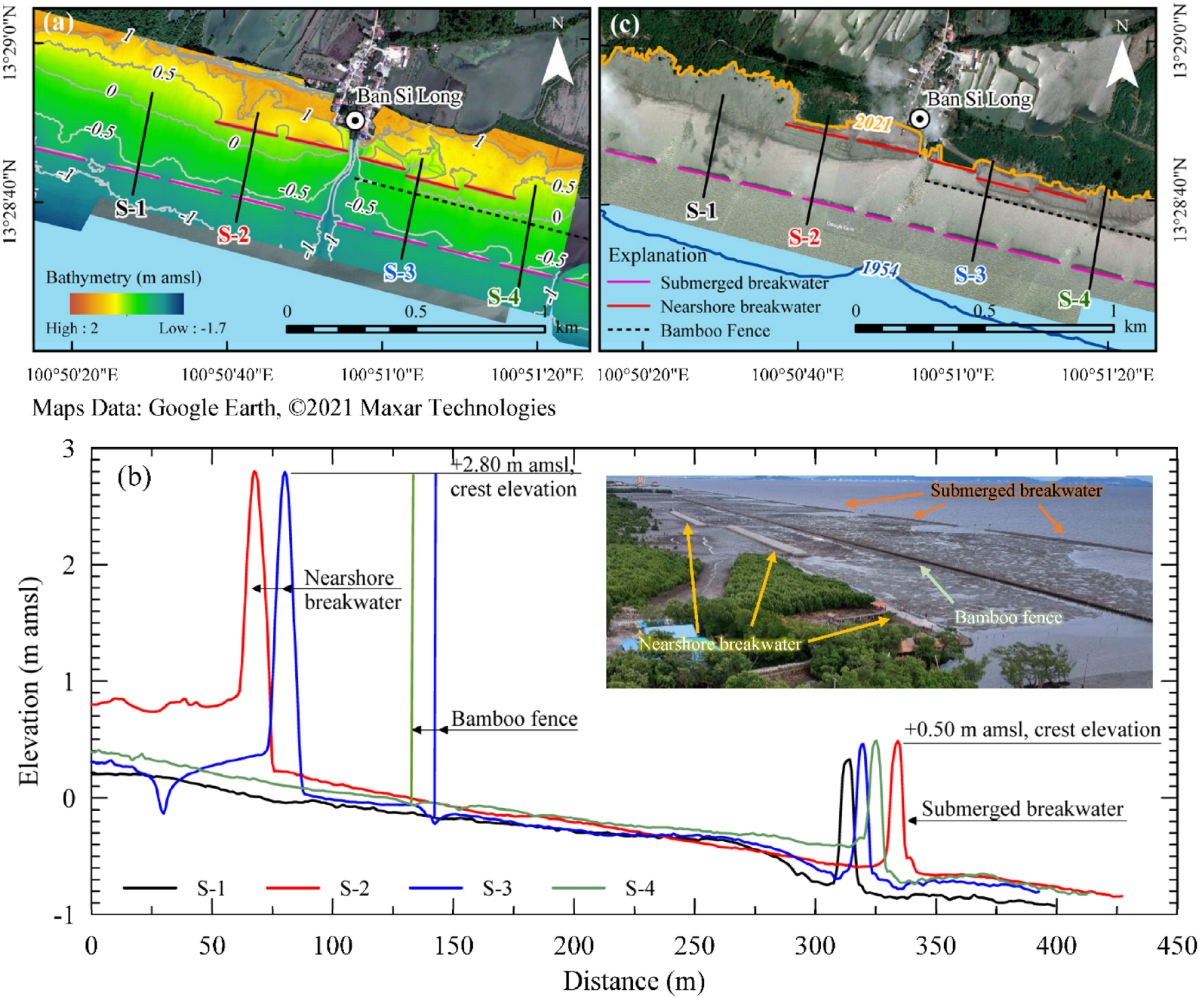
(**a**) LiDAR observation digital terrain model generated from the LiDAR point cloud at Ban Si Long. (**b**) Cross-section profiles of the shore and seabed and coastal protection structures location at Ban Si Long. The top-right photo shows the alignment of the structures and seafloor east of Ban Si Long. (**c**) The coastal protection structures were installed in the eroded area (between shorelines in 1954–2021). The satellite images of a and c were downloaded from Google Earth Pro (©2021 Maxar Technologies, https://www.google.com/earth), and the maps were created using ArcMap software version 10.6 ( © ESRI, https://desktop.arcgis.com). The photograph was taken by author.

A remote sensing technique using LiDAR with an Unmanned Aerial Vehicle (UAV) was used to measure the seabed elevation in front of and behind each coastal structure. The elevation difference between them represents the sediment deposition depth since installation. The sediment deposition rate can thus be estimated from the net sediment deposition depth divided by the time span between the construction and the LiDAR survey. Figure 3a depicts the seabed elevation at Ban Si Long village (red rectangular in Fig. 1a; village #4 in Fig. 1b) where the nearshore and submerged breakwaters and bamboo fence overlapped to some degree. The seabed profiles of four sections (S-1–S-4 in Fig. 3a) are shown in Fig. 3b. The S-1 section shows the seabed response due to the installation of rubble-mound-submerged breakwaters, while S-2 presents the seabed response to nearshore and submerged breakwaters. The effects of the nearshore breakwater, submerged breakwater, and bamboo fence on the seabed level are shown in S-3, and the effects of the bamboo fence and submerged breakwater can be seen in S-4.

The S-1–S-4 profiles (Fig. 3b) show the effect of the rubble-mound-submerged breakwater with a height of 1 m after six years of construction. The structures were constructed 350 to 420 m from the initial shoreline. The seabed differences in front of and behind the structures vary from 20 to 35 cm with an average of 30 cm (5 cm/y). The bed slope behind the structure ranges between 1:300 and 1:600, while the slope in front of the structure ranges between 1:500 and 1:875.

The 3-m high bamboo fence was installed approximately 150–250 m from the initial shoreline in 2015. The effects of a bamboo fence on trapping sediment can be evaluated from profiles S-3 and S-4 (the blue and green lines in Fig. 3b). The analysis of the bottom level reveals that the seabed elevation at the back of the fence differs only 5–8 cm (< 1.3 cm/y) from the bed level at the front of the fence. Additionally, the slopes observed behind and in front of the structure vary from 1:350 to 1:600 and 1:500 to 1:600, respectively.

The S-2 and S-3 profiles in Fig. 3b show the different levels of seabed affected by the nearshore breakwaters during 2014–2019. The structures were constructed at about 70–100 m from the initial shoreline, with the height varying between 2.5 and 2.8 m. The bed levels behind the structure were about 35–70 cm higher from those in front of the breakwater (17–23 cm/y). The seabed gradient found at the front of the structure was about 1:300 and 1:550, while the bottom slope behind the structure reduced to 1:640–1:1200.

## Discussion

Since the ECPD muddy coastline has the greatest shoreline loss rate in the world^38^, many coastal protection measures, both grey and green types, have been applied along the coastline during the past three decades. Seawalls, one of the hard solutions commonly used to stabilize sandy beaches, was first used to stabilize the ECPD coast. According to our horizontal shoreline change results, the seawall constructed near the Chao Phraya River mouth in 1994 has successfully stabilized about 23% of the ECPD coastline. The remaining unprotected coastline still underwent severe shoreline recession during 1954–2013 due mainly to land subsidence caused by groundwater extraction^14,28^. This study evaluated the efficacy of the grey and green/nature-based solutions applied along the ECPD coastline using two approaches. The horizontal shoreline change approach was used to assess each solution's effectiveness on shoreline stabilization. The success of each solution was evaluated by comparison between pre-and post-construction shoreline change rates. However, the different engineered structures that are being compared do not necessarily occur at the same location. Therefore, they are subject to differences in other environmental conditions including sediment supply, land reclamation, mangrove colonization, and subsidence due to groundwater extraction. These factors can also directly affect sedimentation rate behind these structures and introduce both spatial and temporal uncertainties in our assessment of engineered defense effectiveness. To minimize these effects, we emphasized an area just west of the Bang Pakong River (Ban Si Long, #4 in Fig. 1b) where the grey and green structures have maximum spatial overlap. We used the vertical seabed level change approach to evaluate the effectiveness of each solution on sediment trapping. The comparison between ground elevation in front of and behind each structure derived from LiDAR data was used to calculate sediment deposition depth behind the structure. By dividing the deposition depths with the known period between the construction and LiDAR observations, the vertical deposition rates (cm/y) due to the structure can be estimated. Based on the results from this study, the effectiveness of each structure is discussed as follows.

**Figure 4:**
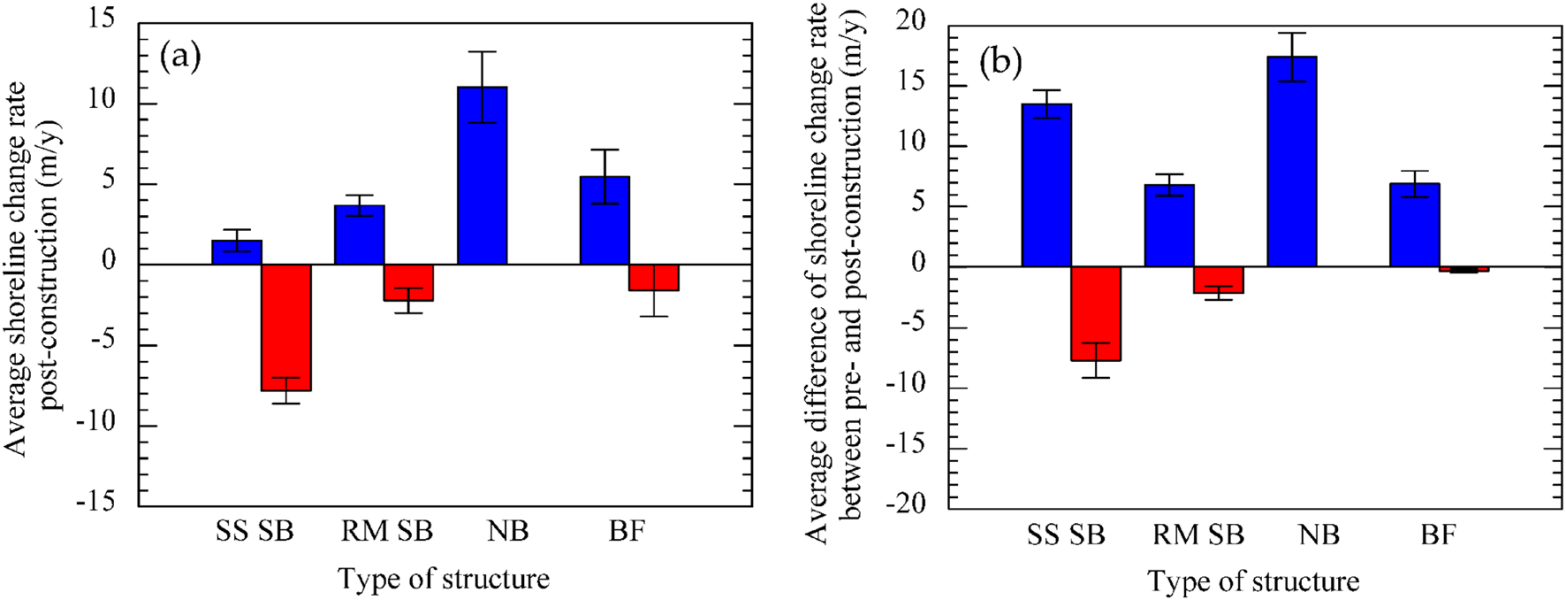
(**a**) Average shoreline change rates after construction of each structure. (**b**) Average differences in shoreline change rates before and after construction of each structure. NB is a nearshore breakwater, BF is a bamboo fence, and SS-SB and RM-SB are sand-sausage-submerged breakwater and rubble-mound submerged breakwater, respectively.

Submerged breakwaters, a common hard solution, were applied along a 23-km shoreline of the mid-portion of the ECPD coastline during 2004–2005. Sand sausages were used to overcome the sinking of the structure using heavy materials, especially rock. Table 2 and Fig. S1a indicate that only 12% of protected shorelines became stable or showed accretion, while the remaining 88% still experienced shoreline recession with an average shoreline accretion and retreat rate after the construction of about 2 m/y and − 8 m/y, respectively (Fig. 4a). Saengsupavanich^35^ also reported that sand sausages were ineffective in protecting the shore during high tides because waves could overtop the crest of the structure and eventually erode the coastline. However, the data of Table 2 shows that the average shoreline retreat rate after construction was reduced to half of that before deploying the sand sausages. Figure 4b, which illustrates the performance of sand sausage averaged from the differences of shoreline change rates between pre- and post-construction also supports this view. Based on a plot of different rates of shoreline changes between pre-and post-construction of each structure versus the structure's distance from the initial shoreline (Fig. S1b), sand sausages placed between 40 and 450 m from the initial shoreline resulted in 64% of the eroded shoreline having a lower shoreline retreat rate than before construction. Moreover, 78% of those sand sausages successfully reduced the shoreline retreat rate of 1–20 m/y with an average rate of 13.5 m/y (Fig. 4b). However, the remaining 36% of sand sausages located along the coastline near the BPK River did not ameliorate the shoreline recession. Even though most of them were installed at about 10–150 m from the initial shoreline, the shoreline retreat rate still increased with an average rate of about – 8 m/y (Fig. 4b).

Since the shoreline responded to the existing sand sausages without a systematic trend, other factors may outweigh their effectiveness. Because a high rate of land subsidence was reported in this area during 1992–2015^14,38^, the unsuccessful use of sand sausages on coastal stabilization may be caused by that very high rate of land subsidence (up to 6 cm/y). Another possible factor responsible for the ineffectiveness of sand sausage during 2002–2010 can be the settlement of the structure combined with sand leakage from their geocontainers resulting in lowering the level of the breakwater’s crest^35^. As sand sausages can only function for four to five years, they might not be active as designed, and a higher rate of shoreline recession may result.

Beside the short-life span (only 4–5 years) and insignificant success on shoreline protection, the deterioration of geotextile sandbags was another issue causing several serious environmental problems^34,35^. The leakage of sand contaminating the muddy beach affects the coastal ecology, causing the death of aquatic creatures living on the muddy coast and mangroves^35^. In 2015, the Marine Department replaced the sand sausage with a rubble mound to reduce the negative environmental impacts. Although the rubble mound was installed on top of the sand sausages with the same crest level as the sand sausage construction, the results in Table 2 and Fig. 4a indicate that the effectiveness of the rubble mound breakwater was obviously higher than the sand sausage. Additionally, the LiDAR data also confirmed the success of rubble-mound breakwater in trapping sediment with an average rate of about 5 cm/y. Based on the historical image taken in 2015 during the northeast monsoon, the construction of submerged breakwaters likely induces sediment deposition behind structures by trapping offshore sediment transport, as shown in Fig. S2. Regarding Fig. S1a, the shoreline seems to be successfully stabilized where the submerged breakwaters were placed at less than 450 m from the shoreline. Still, no strong correlation between the degree of success in protecting the coastline and the distance from the structure to the initial shoreline was observed. Figure 4a indicates that the rubble-mound-submerged breakwaters caused higher rate of shoreline advance and lower rate of shoreline retreat than the sand sausage, even though they have similar functions. The rubble-mound breakwater's higher effectiveness than sand sausage may be related to the relatively lower rate of land subsidence, which varied only between 1.0 and 2.5 cm/y during 2000–2015^14^ combined with the durability of the rubble mound (> 10 y). Furthermore, no negative environmental issues have been reported after construction. In contrast, local villagers living along the ECPD coastline informed us that an accretionary area formed behind the mound, which used to be characterized by erosion. Moreover, the area has been recolonized with aquatic animals, especially oysters, mudskippers, and crabs.

Bamboo fences are considered a nature-based green measure suitable for coastal wetland protection^20,39,40^. These structures have been applied to the ECPD coast since 2009 by the DMCR. Several studies suggested that bamboo fences could effectively decrease wave energy and accelerate sediment deposition behind the structure. Based on a study by Mai Van C, et al.^20^, it was found that the bamboo fence can reduce wave height up to 50% depending on the water depth and wave steepness, and the sedimentation depth can increase up to 60 cm after 10 months. The shoreline analysis in our study also shows that bamboo fences are a successful measure for protecting the ECPD shoreline since it can stabilize and increase accretion shorelines of about 74% of its protected shoreline (Table 2). However, the remaining 26% located near the BPK River mouth still underwent shoreline recession.

Even though a bamboo fence was applied along part of the shoreline at 50–200 m offshore since 2009, the high accretion rate behind the bamboo fence was observed only along the coastline protected by revetments (Fig. S3). The coastline without revetments still migrated landward at − 4 to − 29 m/y. A high shoreline accretion rate of more than 20 m/y was found in this mangrove coastline during 2014–2016 (at least three years after the installation). Even though most bamboo piles completely broke down in 2015, mangroves kept colonizing seaward behind and in front of the fence (Fig. S2). However, the LiDAR data observed at Ban Si Long indicates no apparent difference in the seabed elevation between the back and front of the structure (Fig. 3b). With an estimated deposition rate behind the bamboo fence of less than 1.3 cm/y, the bamboo fence is not effectively trapping sediment. Therefore, high average accretion rate along the bamboo-fence-protected coastline (Fig. 4a) may be dominated by other factors, especially land subsidence, which typically alters the shoreline change patterns.

Land subsidence along the ECPD coastline due to excess groundwater pumping has been reported since 1984^14^ (Fig. S4). The subsidence rate increased from about 5 cm/y in 1985 to 6.3 cm/y in 1995. The sinking rate decreased to less than 2.5 cm/y after 2011 and less than 1 cm/y after 2015. For this reason, even though the Department of Marine and Coastal Resources (DMCR) started to install bamboo fences in 2011, the noticeable accretion rate was observed after 2014 because the sediment deposition rate behind the structure was higher than the rate of land subsidence. The sedimentation rate of the bamboo fence was less than 1.3 cm/y, less than the subsidence rate. Therefore, the shoreline accretion behind the bamboo fence may be induced by sedimentation of the submerged breakwaters (5 cm/y) placed in front of the bamboo fences (Figs. S2b and 3). In 2019, bamboo fences were applied near the BPK River mouth, where land subsidence may dominate the shoreline retreat in this area^14^, yet the recession of the mangrove shoreline has persisted to present. With a low sedimentation rate combined with the short-life span, bamboo fences are not the best choice for stabilizing shorelines when the subsidence rate is higher than the sediment deposition rate.

Based on the nature-based solution concept, a bamboo fence is used to assist in the natural regeneration of mangroves by trapping sediments. After successful regeneration of mangroves, the mangrove will become stable in several years as they trap sediment by themselves. Then the existence of a bamboo fence becomes unnecessary. However, on the ECPD coast, the use of bamboo fences has caused an unforeseen environmental impact. Unlike the Viet Nam coast reported by Mai Van C, et al.^20^, the ECPD coast has a high rate of land sinking and a low sedimentation rate; then, mangrove recolonization commonly does not regenerate during the first few years. After 2–3 years of bamboo fence construction, the deterioration of bamboo broke the fence into two parts, resulting in the structure being ineffective in reducing wave energy and trapping sediment. Meanwhile, the sediment trapped during the first few years has been moved offshore during the high tide and high wave conditions before the mangrove can regenerate. The broken upper part of the fences became coastal debris along the coastline (Fig. S5), while the remaining embedded part (Fig. S6) obstructed coastal access and deteriorated the coastal landscape. Even though the bamboo fencing has been disapproved by local communities living along the ECPD coastline since 2013 due to its negative environmental and social impacts^35^, bamboo fences are still considered a green/nature-based solution for shoreline protection in Thailand.

Because of the failure and negative impacts of the green solution, the local governments started to construct nearshore breakwaters, a grey solution, in 2015. The breakwaters were built at 40–280 m far from the coastline, and they have successfully reclaimed the coastline. The results from this study indicate that nearshore breakwaters can stabilize the ECPD shoreline with no shoreline retreat behind these breakwaters, and shoreline accretion was found within a few years after construction (Fig. S7). As the structures were typically constructed near the coastline with a height greater than 1.5 m above shoreline level, the structures function not only as wave energy dissipators but also as sediment trappers. Figures 3a, b reveals that the nearshore breakwaters constructed at Ban Si Long effectively trap onshore-offshore sediment resulting in a sediment deposition rate of 17–23 cm/y behind the structure. Similar to the rubble-mound-submerged breakwater, no adverse environmental impacts have been issued regarding the construction of the nearshore breakwater. Our study found that the proper location for nearshore breakwater construction should be between 100 and 250 m offshore. If the structure is installed closer than 100 m, it will obstruct the colonization of the mangrove shoreline (Figs. S7a, b), resulting in less accretion area.

Based on sedimentation and shoreline change rates, the nearshore breakwater seems to be the most successful structure among the shoreline protection structures used to date along the ECPD coastline. Even though results from shoreline change analysis indicated that the submerged breakwater has the lowest effectiveness on land reclamation, the surface elevation map (Fig. 3a) shows that the submerged breakwaters have resulted in higher sedimentation rates than bamboo fences. Since nearshore sediment processes in the study area are dominated by onshore-offshore sediment transport^14^, the submerged breakwater can effectively trap the sediment transported offshore even if it has less effectiveness on dissipating wave energy compared to nearshore breakwaters and bamboo fences. These defenses, which typically are installed near the coastline with their height above mean high-water, effectively dissipate wave energy and trap sediment resulting in higher sedimentation rates behind the structure. However, sediment deposited behind bamboo fences is normally moved offshore due to tidal currents after the deterioration of these fences causing low rates of sedimentation. As the bamboo fence seems to have higher effectiveness in stabilizing the shoreline than submerged breakwaters if the efficacy is based on the horizontal change of the shoreline (Figs. 3c, 4a, and S3), the bamboo fence has been applied along Thailand’s coastlines as a nature-based solution instead of seawalls and breakwaters. However, the shoreline change rates can be biased by shoreline proxies and other environmental effects such as land subsidence and human activities. The effectiveness of different structures should be analyzed based on the surface elevation data to get unbiased information for proper coastal management.

## Conclusions

We evaluated the effectiveness of both grey solutions (submerged and nearshore breakwaters) and a green solution (bamboo fences) used for protecting the ECPD low-lying-muddy shoreline using shoreline change analysis. Moreover, ground elevation data from the LiDAR survey performed just west of the BPK coastline where there is considerable overlap of the grey and green structures were also used in the assessment of engineered structure efficacy to minimize spatial and temporal uncertainties due to the differences in environmental conditions such as sediment supply, land reclamation, and land subsidence. The analysis of historical shoreline changes during 1954–2021 revealed that nearshore breakwaters, a typical grey solution, successfully reclaimed the coastline with a sedimentation rate behind the structure of 17–23 cm/y when the structure was installed 100–250 m from the shoreline. The 1-m high sand-sausage-submerged breakwaters were ineffective at stabilizing the shoreline during 2002–2010 due to the influence of land subsidence induced by over-pumping groundwater. Additionally, the sand sausage approach lasts for only a few years due to the deterioration of the covering materials. Furthermore, contamination of sand in the muddy environment may impact the ECPD ecology. The rubble-mound-submerged breakwaters replaced the sand sausages during 2014–2015. Without land subsidence, these submerged breakwaters can stabilize the shoreline with a deposition rate of about 5 cm/y, with no apparent ecological effects. Meanwhile, shoreline analysis indicates that bamboo fences, a popular green/nature-based solution, successfully reclaimed the mangrove-based shoreline in this area. In contrast, the LiDAR data analysis showed that these fences trap the sediment behind the structure at less than 1.3 cm/y. Even though the bamboo fence approach has been used to stabilize muddy mangrove coastlines in many countries as a green solution, the bamboo generally decomposes within 2–3 years after installation. The decomposed bamboo eventually becomes sea debris, causing coastal environmental problems along the coastline.

## Methods and Study Area

### Study area

The ECPD is a low-lying muddy mangrove beach located in the upper Gulf of Thailand (Fig. 1). The coastline extends from the CPY River mouth to the BPK River mouth, about 42 km in length. The sediment that formed the ECPD was derived from the CPY and BPK Rivers, which mainly contain silty or sandy clay and silty clay with organic materials, respectively^38,41^. The climate in the ECPD is dominated by the northeast (NE) monsoon from November to April (dry season) and the southwest monsoon (SW) from May to October (wet season) ^14,42^. The sea climate in this area is generally calm with a significant wave height of less than 0.5 m^42^, while high wave conditions with a significant wave height greater than 2.5 m occurs during the SW monsoon^43^. Based on hydrographic data observed by National Research Council of Thailand during 1997–2000, sea temperatures in this region varies between 16–32 °C with an average temperature of 27.3 °C. The wind and wave direction mainly comes from the north (N) and south (S) during the NE and SW monsoons, respectively^44^. The eastern Chao Phraya Delta is considered an intertidal zone with an average tidal range of 1.5 to 2.5 m during the neap and spring tides, respectively^38^, causing tidal currents in the N-S direction with average speeds of 0–0.8 m/s^44^.

The CPD has been reported as a severe eroding coastline worldwide, with an average shoreline retreat rate during 1954–2019 of about − 10 and − 7 m/y in the western Chao Phraya Delta (WCPD) and eastern Chao Phraya Delta (ECPD) coastlines, respectively^38^. This delta's substantially high rate and rapid shoreline retreat was mainly caused by land subsidence due to excess groundwater extraction^14^. While the WCPD is occupied primarily by aquacultural activities, the ECPD coast hosts the largest industrial complex in the country. Various coastal protection measures have been applied along the ECPD to protect the major commercial and economic areas.

### Shoreline change horizontal analysis

Historical shoreline positions between 1954 and 2021 were obtained from aerial photographs and satellite imagery. The aerial photographs (1954, 1996, 2002) were obtained from the Royal Thai Survey Department (RTSD), and the satellite images (2010–2021) were provided by Maxar Technologies, CNES, and Airbus satellite data using Google Earth Pro software (version 7.3). To eliminate image distortion, ArcGIS version 10.5 was used for georeferencing all images with the orthophotograph of 2002. Table 3 presents the details of the aerial photography and satellite imagery data, including image resolution and georeferencing errors estimated for this study. Since mangrove forests and aquaculture ponds bound much of the ECPD shoreline, the edge of vegetation or mangrove, roads, and dikes were used as shoreline proxies^28,30,45–47^.

**Table 3:** Summary of the data used for assessing the shoreline change along the eastern Chao Phraya Delta coastline.

Year	Type of data	Scale (m)	Image resolution (m)	RMSE^2^ (m)	Shoreline position uncertainty (m)
1954	Aerial photograph	1:50,000	4.60	1.72	4.91
1996	Aerial photograph	1:50,000	4.50	1.21	4.66
2002	Orthophotograph	1:4,000	< 0.1	< 0.1	< 0.1
2010	Satellite image^1^	1:700	0.91	0.72	1.17
2011	Satellite image^1^	1:700	0.90	0.82	1.22
2013	Satellite image^1^	1:700	0.90	0.73	1.17
2014	Satellite image^1^	1:700	0.91	0.66	1.13
2016	Satellite image^1^	1:700	0.91	0.59	1.09
2018	Satellite image^1^	1:700	0.91	0.61	1.10
2021	Satellite image^1^	1:700	0.92	0.67	1.15

^1^Download in JPG format at no cost from Google Earth Pro software version 7.3 (https://www.google.com/earth).

^2^RMSE = Root Mean Square Error from georeferencing process.

This study analyzed changes in shoreline movement patterns between 1954 and 2021 to evaluate the ECPD shoreline response to each coastal protection solution. The shoreline change rate between two successive shorelines was calculated using the Digital Shoreline Analysis System (DSAS) version 5.1^14,45–49^. With a 20 m spacing, 2126 transects were generated normal to the baseline along the ECPD coastline. The distance and rate of shoreline change at each transect were calculated using Net Shoreline Movement (NSM) and End Point Rate (EPR) methods, respectively. In shoreline change estimation, several sources of uncertainty can affect the analysis of shoreline change, especially the shoreline position and shoreline change rate uncertainties^50–52^. The positional uncertainty (*U*_*sp*_) typically includes shoreline proxy offset (*E*_*o*_), tidal fluctuation (*E*_*t*_), seasonal variation (*E*_*s*_), rectification error (*E*_*r*_), digitizing error (*E*_*d*_), pixel error (*E*_*p*_), and toposheet survey error (*E*_*ts*_), and can be calculated using Eq. (1)^53–55^. The shoreline positions in this study were obtained from an image file (TIFF format) of aerial photographs and satellite imagery; position uncertainties are then mainly related to the resolution of the materials and orthorectification or georeferencing processes. Then the terms *E*_*o*_, *E*_*t*_, *E*_*s*_, *E*_*d*_, and *E*_*ts*_ can often be neglected^46,51^. Shoreline change rate uncertainty (*U*_*i*_) is typically quantified as the quadrature sum of uncertainties for the shoreline position each year, divided by the number of years between the first and last shoreline^14,51^. An uncertainty of each transect can be calculated from Eq. (2)^51,54,56^. Table 3 summarizes the resolution of image data and root mean square errors (RMSE) from the georeferencing process for the dataset used in this study.

1 $$U_{sp} = \pm \sqrt {E_{0}^{2} + E_{t}^{2} + E_{s}^{2} + E_{d}^{2} + E_{ts}^{2} + E_{p}^{2} + E_{r}^{2} }$$

2 $$U_{i} = \frac{{\sqrt {U_{1}^{2} + U_{2}^{2} + \ldots + U_{n}^{2} } }}{\Delta Y}$$

where *U*_*i*_, *i* = 1,…, *n* is the uncertainty at a given transect for shoreline 1, 2, …, n; n is the total number of shorelines; ∆Y denotes the period between the first and the last year of the shorelines^54^.

### LiDAR technology survey

The classic ground survey usually is ineffective and impractical for measuring ground level on muddy mangrove beaches. We applied an Unmanned Aerial Vehicle (UAV)-Light Detection and Ranging (LiDAR) technology to assess the effectiveness of each coastal protection measure in trapping sediment. LiDAR is a remote sensing method that uses light waves to measure the coordinates of surrounding objects. In November 2021, LiDAR measurements were collected at Ban Si Long (Fig. 1b), where both grey and green solutions were installed. A LiDAR sensor DJI Zenmuse L1 mounted on a UAV, DJI Matrice 300 RTK with a flying level of 100 m above the seafloor at 10 m/s. With 50% overlap between adjacent flight lines, a lidar point cloud with a density of 354 points/m^2^ and 5.3 cm point spacing was produced. The data validation results at Ban Si Long show that the RMSE was 2.3 cm. The sensor supports a maximum of 3 returns that can have more chance of penetrating mangrove forest area to ground level. The details of sensor specification and accuracy can be found at https://www.dji.com/zenmuse-l1/specs.

The collected LiDAR data from a field survey need to be post-processed before producing a study area's digital terrain model (DTM). Three to five checkpoints were placed around the study area on stable ground, such as a road, concrete slab, or firm flat area, to validate the collected data accuracy. The coordinates of these checkpoints were measured using the global navigation satellite system (GNSS) with a real-time kinematic positioning (RTK) survey method. The GNSS device, Stonex S10 (Viale dell'Industria 53 | 20,037 Paderno Dugnano (MI), Italy) has a horizontal accuracy of 8 mm and a vertical accuracy of 15 mm. Elevation data (Z) known from the GNSS survey were used to validate the accuracy of the elevation data obtained from LiDAR measurements. LiDAR uncertainties can be calculated using Eq. (3).

3 $${\text{RMSE}}_{z} = \frac{{\sqrt {\sum {\left( {\Delta Z^{2} } \right)} } }}{m}$$

where *ΔZ* is the difference in checkpoint coordinates according to the reference measurement from the particular point cloud, and *m* is the number of reference checkpoints. The RMSE_z_ was used to adjust the ground elevation of point clouds. The corrected ground points were then used to produce the study area's DTM.

## Supplementary Information

Supplementary Information.

## Data Availability

The datasets used and/or analysed during the current study available from the corresponding author on reasonable request.
