# Supplementary material for: Effectiveness of grey and green engineered solutions for protecting the low-lying muddy coast of the Chao Phraya Delta, Thailand
Source: Sci Rep. 2022 Nov 28;12:20448. doi: 10.1038/s41598-022-24842-x (PMC9705285; doi:10.1038/s41598-022-24842-x)
Supplement: Supplementary file 1 — Supplementary Information. [file 41598_2022_24842_MOESM1_ESM.pdf]

Effectiveness of grey and green engineered solutions for protecting the low-lying muddy coast of the Chao Phraya Delta, Thailand

Warit Charoenlerkthawin[1], Komkrit Bidorn[2], William C. Burnett[3], Jun Sasaki[4], Balamurugan Panneerselvam[5], Butsawan Bidorn[1,2*]

[1]Department of water resources Engineering, Chulalongkorn University, Bangkok 10330, Thailand
[2]WISE Research Unit, Chulalongkorn University, Bangkok 10330, Thailand
[3]Department of Earth, Ocean and Atmospheric Science, Florida State University, Tallahassee, Florida 32306, USA
[4]Department of Socio-Cultural Environmental Studies, The University of Tokyo, Kashiwa 277-8563, Japan
[5]Department of Community Medicine, Saveetha Medical College, SIMATS, Chennai 602-105, India

Supplemental Information

- Fig. S1: Shoreline change rates after construction of each structure versus distance from the initial shoreline and the differences in shoreline change rates before and after construction of each structure.
- Fig. S2: Sediment trapped sediment behind coastal protection structures.
- Fig. S3: Mangroves recolonized seaward across the deteriorated bamboo fence.
- Fig. S4: Shoreline change, land subsidence rates, and groundwater extraction in the eastern Chao Phraya Delta since 1954.
- Fig. S5: Bamboo debris at Ban Si Long after three years from construction.
- Fig. S6: Bamboo fence east of Ban Si Long between Sta.28+540 and Sta.29+500. after 2–3 years from construction.
- Fig. S7: Trapped sediment and recolonizing mangrove behind nearshore breakwaters constructed in 2015.

Supplemental materials

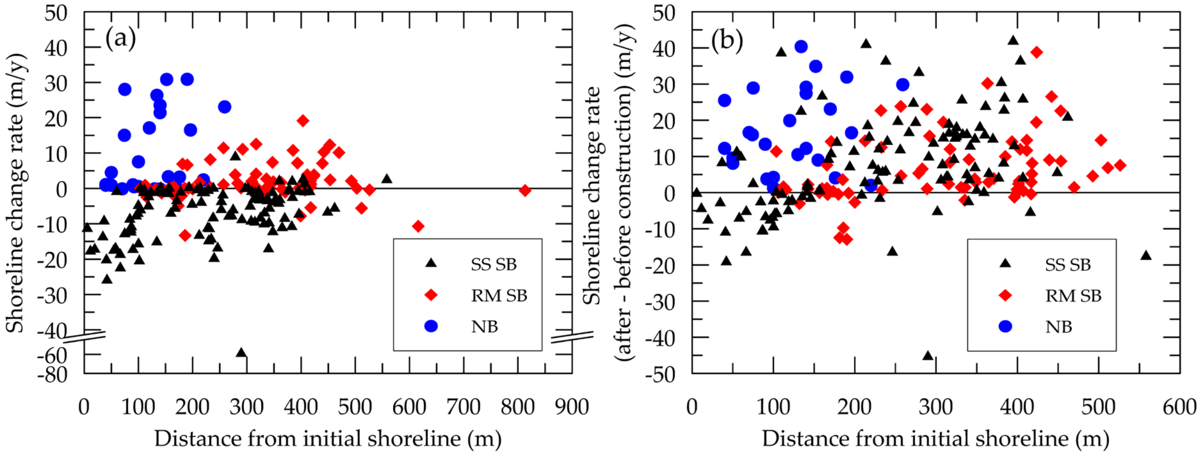

Figure S1. (**a**) Shoreline change rates after construction of each structure versus distance from the initial shoreline. (**b**) Differences in shoreline change rates before and after construction of each structure. NB is a nearshore breakwater and SS-SB and RM-SB are sand-sausage-submerged breakwater and rubble-mound rubmerged breakwater, respectively.

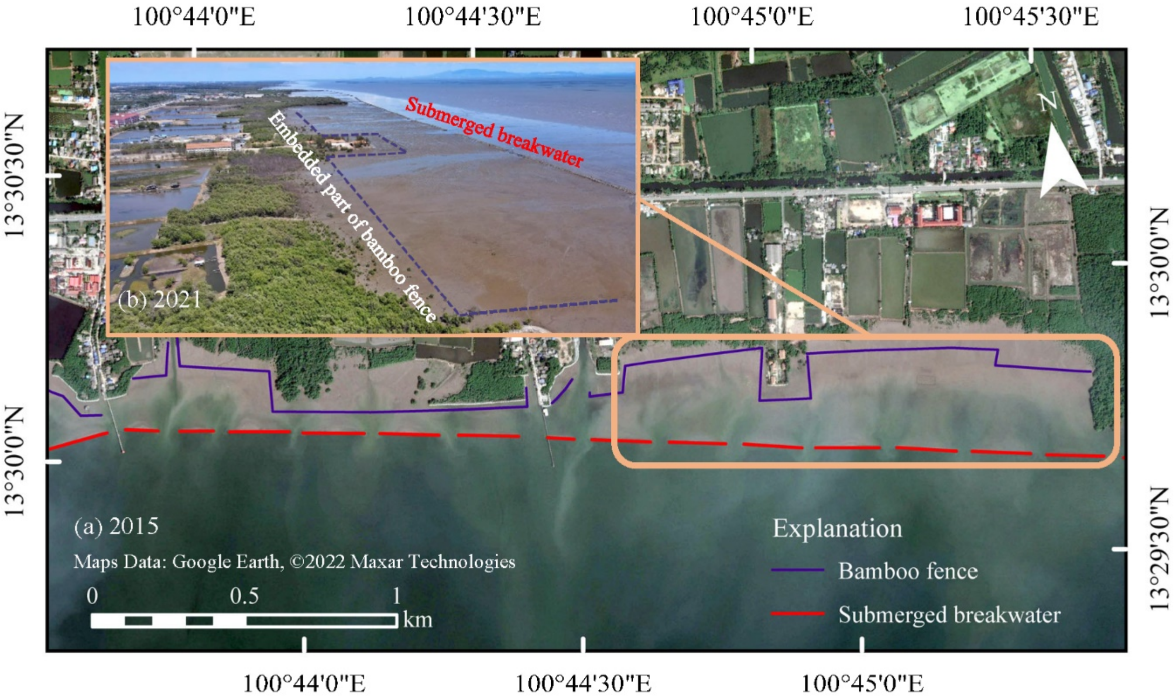

Figure S2. (**a**) Submerged breakwaters between Sta.15+000 to Sta.18+400 effectively trap sediment behind the structure. The satellite image was downloaded in JPG format from Google Earth Pro (©2022 Maxar Technologies, https://www.google.com/earth), and the map was generated using ArcMap software version 10.6 (© ESRI, https://desktop.arcgis.com). (**b**) Sediment trapped by submerged breakwaters. The purple dashed line shows the embedded part of the bamboo fence, which has completely deteriorated since 2015 (The image was taken by author).

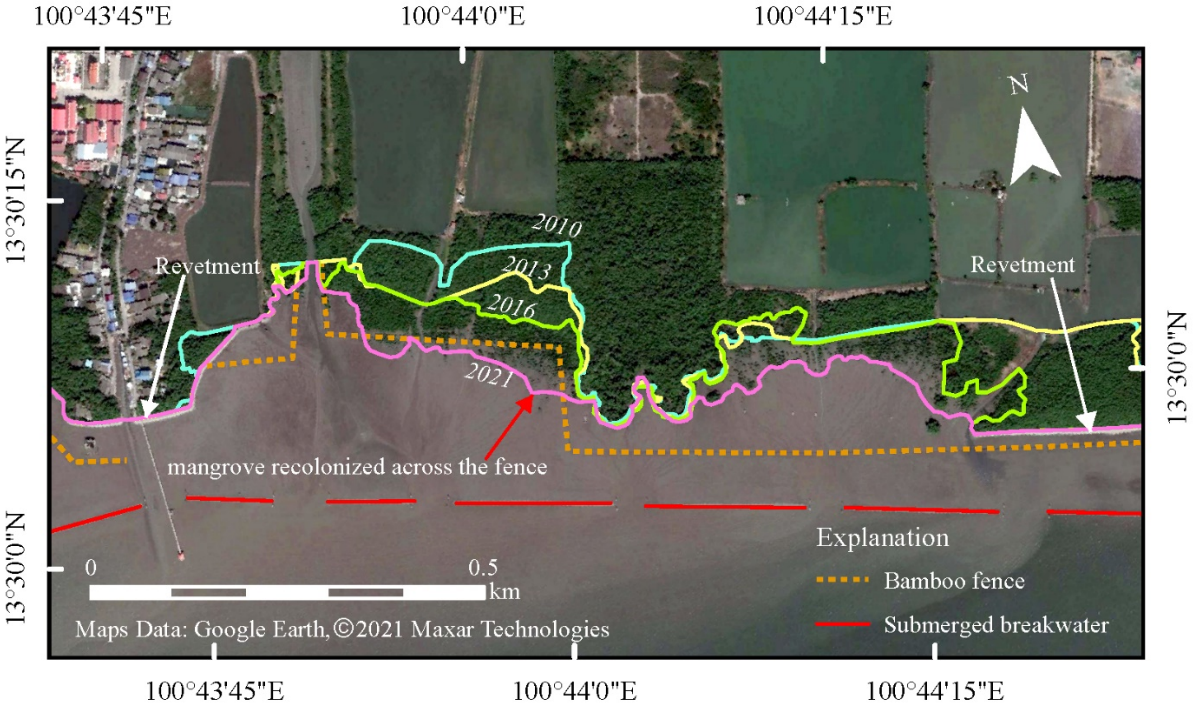

Figure S3. Mangroves recolonized seaward across the deteriorated bamboo fence between Sta.15+040–Sta.16+500. The satellite image was downloaded in JPG format from Google Earth Pro (©2021 Maxar Technologies, https://www.google.com/earth), and the map was created using ArcMap software version 10.6 (© ESRI, https://desktop.arcgis.com).

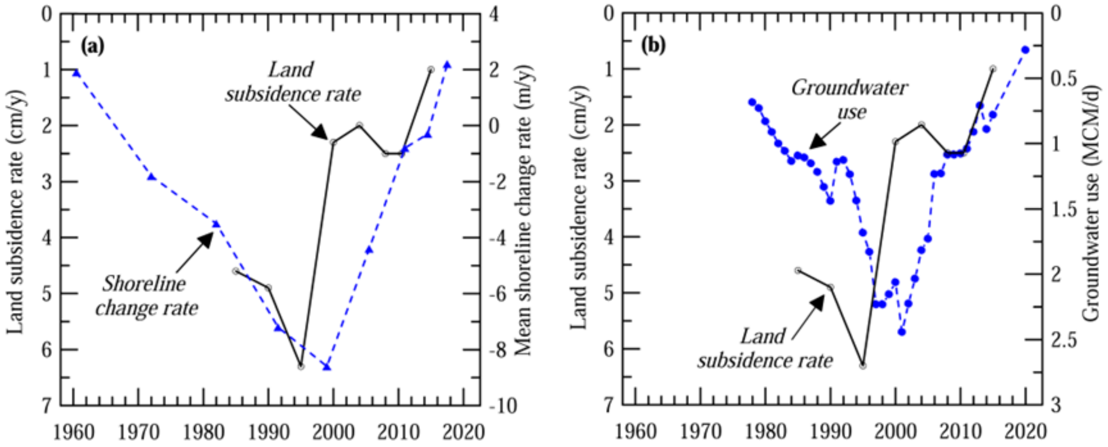

Figure S4. (a) Shoreline change and land subsidence rates in the eastern Chao Phraya Delta since 1954. **(b)** Plot of groundwater extraction in the lower Chao Phraya plain during 1962–1992 together with rates of subsidence in the same area studied by Sok et al.[14]

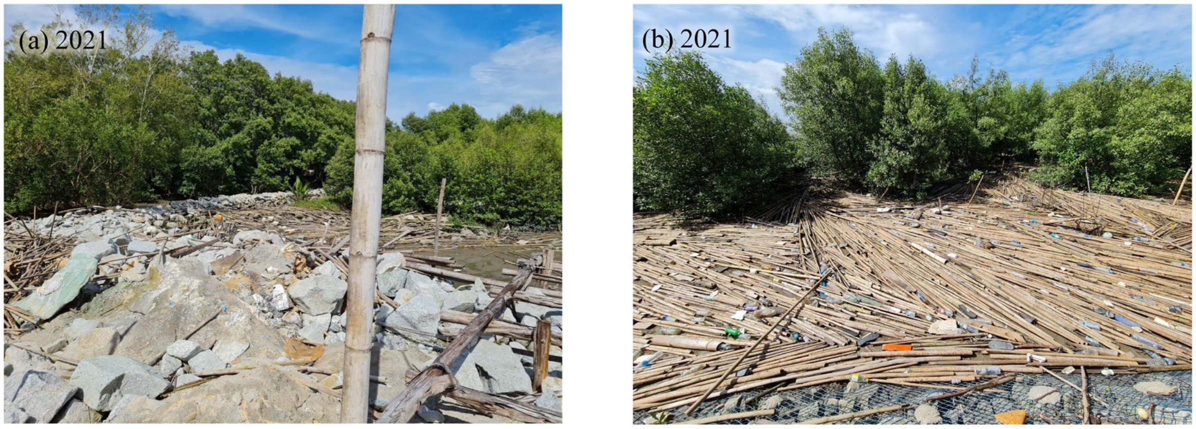

Figure S5. (**a**) Debris bamboo east of Ban Si Long after three years from construction. (**b**) Bamboo debris west of Ban Si Long three years after construction (The images were taken by author).

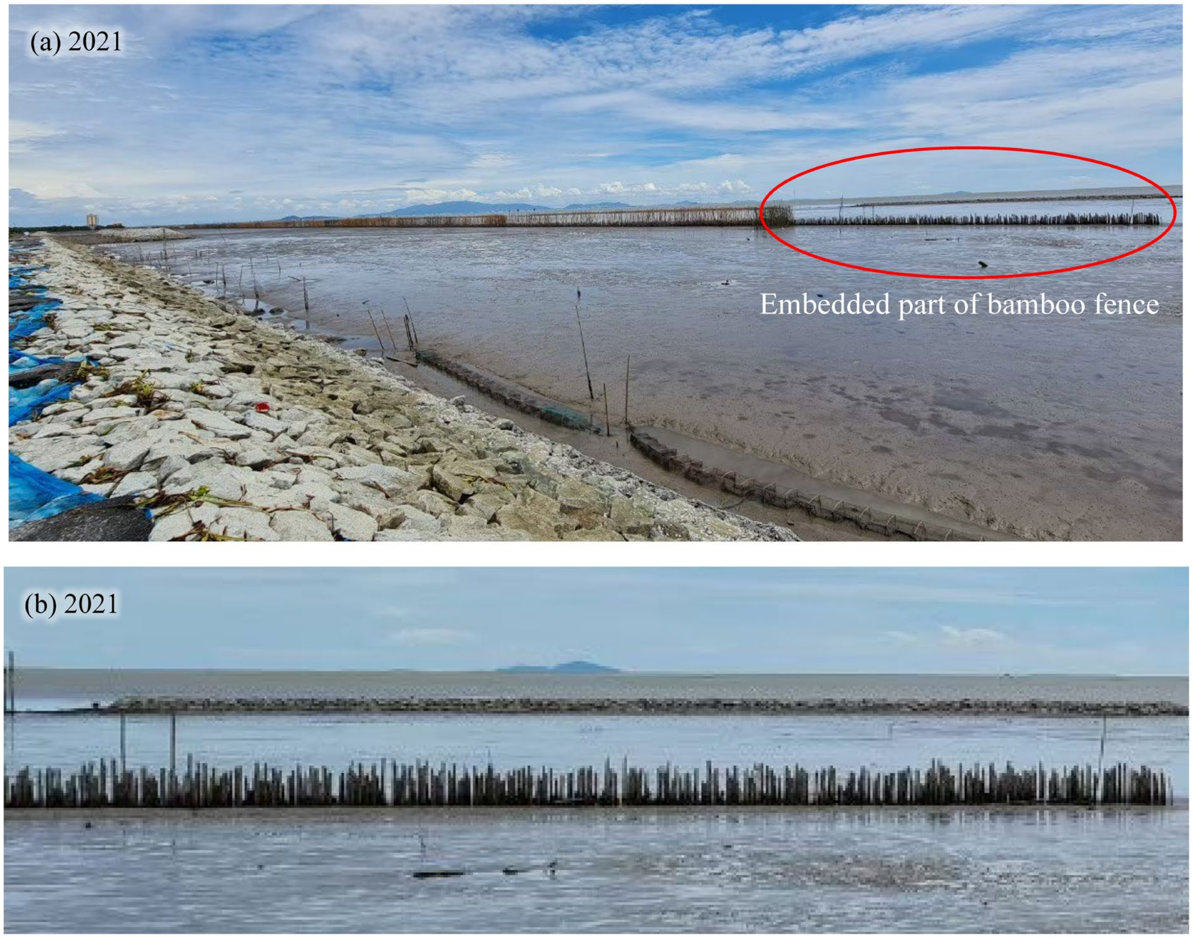

Figure S6. (**a**) Bamboo fence east of Ban Si Long between Sta.28+540 and Sta.29+500. As the bamboo normally breaks into two parts after 2–3 years from construction, the red ellipse shows the embedded part of the bamboo fence. (**b**) The embedded part of the fence seems too short to dissipate the wave energy during high tide and obstructs coastal access (The images were taken by author).

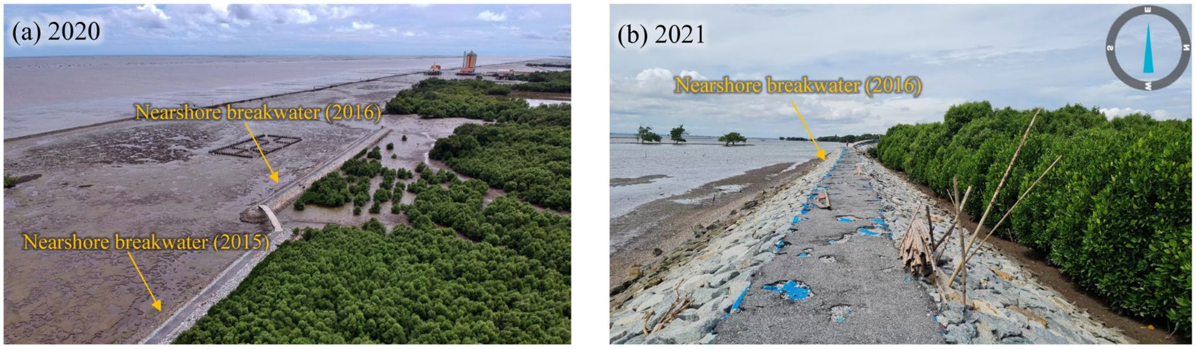

Figure S7. (**a**) Nearshore breakwaters constructed in 2015 and 2016 at Ban Khlong Hong Thong coastline (Sta.31+860–Sta.32+220) effectively trapped sediment behind the structures resulting in mangrove rehabilitation. On the other hand, these structures obstructed mangroves from colonizing further seaward. (**b**) Mangroves fully occupied the lee side of the nearshore breakwater constructed in 2016 within one year (2020–2021). The structure trapped approximately 1-m high sediment after five years after construction (The images were taken by author).
